# Supplementary material for: Modeling glioblastoma heterogeneity as a dynamic network of cell states
Source: Mol Syst Biol. 2021 Sep 16;17(9):e10105. doi: 10.15252/msb.202010105 (PMC8444284; doi:10.15252/msb.202010105)
Supplement: Supplementary file 6 — Source Data for Figure 5 [file MSB-17-e10105-s004.zip › Figure5A_sourcedata/GSEA_3017/hallmarks_stateD.GseaPreranked.1621934542601/HALLMARK_MTORC1_SIGNALING.html]

Details for gene set HALLMARK\_MTORC1\_SIGNALING[GSEA]

|  || Dataset | state23017 |
| Phenotype | NoPhenotypeAvailable |
| Upregulated in class | na\_neg |
| GeneSet | HALLMARK\_MTORC1\_SIGNALING |
| Enrichment Score (ES) | -0.30163518 |
| Normalized Enrichment Score (NES) | -1.3222373 |
| Nominal p-value | 0.15198238 |
| FDR q-value | 0.26960313 |
| FWER p-Value | 0.992 |
Table: GSEA Results Summary

  

Fig 1: Enrichment plot: HALLMARK\_MTORC1\_SIGNALING      
 Profile of the Running ES Score & Positions of GeneSet Members on the Rank Ordered List

  

| PROBE | GENE SYMBOL | GENE\_TITLE | RANK IN GENE LIST | RANK METRIC SCORE | RUNNING ES | CORE ENRICHMENT || 1 | SQLE |  |  | 25 | 0.421 | 0.0100 | No |
| 2 | IDI1 |  |  | 34 | 0.382 | 0.0305 | No |
| 3 | PPA1 |  |  | 64 | 0.328 | 0.0309 | No |
| 4 | STC1 |  |  | 67 | 0.322 | 0.0518 | No |
| 5 | LDHA |  |  | 92 | 0.297 | 0.0539 | No |
| 6 | HMGCR |  |  | 141 | 0.262 | 0.0349 | No |
| 7 | PPP1R15A |  |  | 149 | 0.259 | 0.0476 | No |
| 8 | INSIG1 |  |  | 150 | 0.258 | 0.0656 | No |
| 9 | NUP205 |  |  | 165 | -0.251 | 0.0722 | No |
| 10 | MCM2 |  |  | 226 | -0.256 | 0.0435 | No |
| 11 | PSAT1 |  |  | 241 | -0.258 | 0.0507 | No |
| 12 | PIK3R3 |  |  | 277 | -0.261 | 0.0418 | No |
| 13 | ATP2A2 |  |  | 433 | -0.277 | -0.0593 | No |
| 14 | NAMPT |  |  | 450 | -0.279 | -0.0522 | No |
| 15 | IFRD1 |  |  | 469 | -0.282 | -0.0465 | No |
| 16 | PSPH |  |  | 582 | -0.296 | -0.1128 | No |
| 17 | PHGDH |  |  | 662 | -0.312 | -0.1524 | No |
| 18 | ADD3 |  |  | 663 | -0.312 | -0.1306 | No |
| 19 | FDXR |  |  | 703 | -0.320 | -0.1385 | No |
| 20 | PGM1 |  |  | 715 | -0.322 | -0.1246 | No |
| 21 | PFKL |  |  | 771 | -0.337 | -0.1438 | No |
| 22 | MCM4 |  |  | 783 | -0.339 | -0.1286 | No |
| 23 | TUBG1 |  |  | 917 | -0.381 | -0.2054 | No |
| 24 | RPA1 |  |  | 946 | -0.393 | -0.1997 | No |
| 25 | VLDLR |  |  | 1004 | -0.416 | -0.2149 | No |
| 26 | PLK1 |  |  | 1082 | -0.460 | -0.2426 | No |
| 27 | TFRC |  |  | 1159 | -0.521 | -0.2652 | Yes |
| 28 | CCNF |  |  | 1160 | -0.522 | -0.2287 | Yes |
| 29 | DHFR |  |  | 1197 | -0.576 | -0.2164 | Yes |
| 30 | PLOD2 |  |  | 1215 | -0.615 | -0.1866 | Yes |
| 31 | AURKA |  |  | 1271 | -0.763 | -0.1761 | Yes |
| 32 | RRM2 |  |  | 1295 | -0.933 | -0.1287 | Yes |
| 33 | CDKN1A |  |  | 1306 | -1.046 | -0.0634 | Yes |
| 34 | IGFBP5 |  |  | 1307 | -1.052 | 0.0101 | Yes |
Table: GSEA details [plain text format]

  

Fig 2: HALLMARK\_MTORC1\_SIGNALING: Random ES distribution      
 Gene set null distribution of ES for **HALLMARK\_MTORC1\_SIGNALING**
